# Use of Balloon Occluded Retrograde Transvenous Obliteration (BRTO) for Treatment of Gastric Varices: A Narrative Review

**DOI:** 10.7759/cureus.38233

**Published:** 2023-04-28

**Authors:** Anum Khakwani, Manan Trivedi, Maham Afzal, Puneet Kahlon, Khola ., Parakh Patel, Punith Chowdary Chirumamilla, Rimsha R Vohra, Rani Ratheesh, Midhun Mathew, Zain U Abdin, Zahra Nazir

**Affiliations:** 1 Internal Medicine/Gastroenterology, Nishtar Medical University, Multan, PAK; 2 Department of Surgery, Seth Gordhandas Sunderdas Medical College and King Edward Memorial Hospital, Mumbai, IND; 3 Medicine and Surgery, Shalamar Medical and Dental College, Lahore, PAK; 4 Medicine, American International Medical University, Gros Islet, LCA; 5 Internal Medicine, Shalamar Medical and Dental College, Lahore, PAK; 6 Internal Medicine, Guntur Medical College, Guntur, IND; 7 Internal Medicine, Dow University of Health Sciences, Karachi, PAK; 8 Internal Medicine, Dr MGR Medical University, Tamilnadu, IND; 9 Department of Internal Medicine, Pennsylvania Hospital, Philadelphia, USA; 10 Medicine, District Head Quarter Hospital, Faisalabad, PAK; 11 Internal Medicine/Clinical Research, California Institute of Behavioral Neurosciences & Psychology, Fairfield, USA

**Keywords:** gi endoscopy, transjugular intrahepatic portosystemic shunt (tips, gastric varices, hemorrhage, balloon-occluded retrograde transvenous obliteration (brto)

## Abstract

Gastric Varices occur as a result of portal hypertension. Balloon Retrograde Transvenous Obliteration (BRTO) is a modality for managing gastric varices. The ultimate goal of this review is to promote the broader adoption of BRTO in managing gastric varices and to promote further research to improve patient outcomes.

Before this study, an electronic literature search was undertaken based on identified concepts, keywords, and other pertinent descriptions. Search databases were developed and included “Gastric varices” AND “BRTO” OR “intervention” OR “treatment” OR “procedure” OR “glue” OR “adhesive”.

The databases selected and thoroughly searched were PubMed, Cochrane Library and ScienceDirect. Following the first search, 274 articles were found in total. By applying inclusion criteria of full-text articles and a period of fewer than five years, the database was reduced to 37 articles, which was then further filtered to include only articles on adults over 19 years old, leaving a total count of 17 articles.

BRTO is a relatively simple procedure to perform once the essential skill is attained and helpful in both emergency and elective management of gastric varices. Its use still needs to be improved by the unavailability and lack of skills. However, there are side effects associated with BRTO as it causes elevation of portal hypertension, recurrent bleeding, hemoglobinuria and pain post procedure. This review emphasizes the need for further research in this field, focusing on refining patient selection criteria, improving the technical aspect of the procedure and enhancing long-term outcomes.

## Introduction and background

Cirrhosis is more prevalent in developed countries, causing significant illness and death. While it ranks as the 14th leading cause of mortality globally, it is fourth in central Europe [1]. Cirrhosis is a chronic liver disease that can lead to various complications, including the development of gastroesophageal varices. Gastrointestinal bleeding is often encountered in patients with cirrhosis, with an incidence of 85% in Child Class C versus 45% in Child Class A [2]. Patients in Child Class C also have up to 30% mortality risk compared to other classes [3]. Nearly half of the patients diagnosed with liver cirrhosis have already developed gastroesophageal varices, which can lead to variceal bleeding. Variceal bleeding is a significant concern, with a 5% risk of bleeding in small varices and a 15% risk in medium to large varices [4]. 

Gastric varices refer to a cluster of blood vessels in the stomach's mucosal or submucosal layer. These vessels are part of a more extensive shunt network connecting the portal and systemic circulation. While the prevalence of gastric varices in patients with portal hypertension ranges from 17% to 25%, it is significantly lower than that of oesophagal varices, around 85% [5]. The risk of bleeding is higher in oesophagal (64%) than in gastric varices (25%), but gastric varices (GV) bleed profusely, leading to significant blood loss and increased mortality [2]. With a 35-90% risk of re-bleeding in gastric varices, a superior treatment modality with higher efficacy is crucial in increasing the survival rate of patients [6]. Several modalities are in practice for the treatment of gastric varices, including endoscopic (cyanoacrylate glue and EUS guided coil), radiological (balloon occluded retrograde transvenous obliteration {BRTO}), surgical (shunt), endoscopic injection sclerotherapy and transjugular intrahepatic portosystemic shunt {TIPS}) [7,8]. Shunt surgery is an invasive procedure that may not be suitable for patients with poor liver function. Endoscopic injection sclerotherapy is ineffective for varices in the upper part of the stomach. It is not easy to reach them with the endoscope, and the sclerosing agents may not work effectively due to the fast blood flow in the area. TIPS is an alternative to surgery and sclerotherapy to address these limitations. However, TIPS does not always lead to the regression of gastric varices [9].

Among the different treatment modalities available, BRTO is a minimally invasive technique introduced in 1996 for improved management of GV-related bleeding [10]. In this procedure, a balloon catheter was inserted into the left adrenal vein through the left renal vein by either the transjugular or transfemoral approach. The outflow of the gastro renal shunt is blocked by inflating the balloon, and 5% ethanolamine oleate iopamidol was injected retrogradely [11]. This endovascular interventional radiological technique is commonly used in many countries, particularly Japan, to treat gastric varices effectively and safely in isolation [12].

The management of GVs often involves minimally invasive endovascular treatments BRTO and TIPS. However, the efficacy and outcomes of these treatments can vary depending on individual and institutional factors. Therefore, there is yet to be a clear consensus on whether BRTO or TIPS is the preferred option for managing GVs [13]. Balloon-occluded retrograde transvenous obliteration (BRTO) has emerged as a promising treatment option for gastric varices.

Several ongoing studies have investigated the efficacy and safety of BRTO, leading to a growing body of literature on the topic. In this narrative review, we aim to provide an up-to-date overview of the current knowledge on BRTO and its role in managing gastric varices. We hope to shed light on the benefits through a comprehensive analysis of the existing literature.

## Review

Gastric varices are a complicated medical problem without optimal treatment modalities or standard algorithms. Severe portal hypertension can lead to increased pressure on vessels leading to gastric mucosal changes, including ulcers, erosions and rupture of collaterals leading to excessive bleeding and sometimes hypovolemia, shock and death [11]. Although they infrequently bleed compared to oesophagal varices, they may lead to massive gastric variceal bleeding resulting in hemorrhagic shock and death in 25-55% of cases due to the rapid flow of blood in the varices and the inflow and outflow vessels [10]. The treatment modalities and diagnostic tests are not standardized because the cause of variceal bleeding can differ. Most cases are seen in liver cirrhosis, and some in the non-cirrhotic liver [12]. Various treatment modalities are available for managing gastric varices used as emergency bleeding control or elective obliteration of varices.

Three-lumen, two-capsule tube is an inexpensive primary treatment modality that has rapidly increased effectiveness for achieving haemostasis. However, it cannot be used alone as there is an increased risk of re-bleeding. Three-chamber, two-capsule tube bridging endoscopy and interventional therapy have a very high success rate of haemostasis. However, it requires intubation after the procedure and may not be effective in the long run [13]. Endoscopic variceal ligation (EVL) and endoscopic injection sclerosis (EIS) are beneficial treatment modalities for acute, severe gastric varices bleeding [13]. These modalities are not used together in a combination. They are not preferred for large bleeding varices and are not used in a state of shock or encephalopathy secondary to cirrhosis and bleeding [13]. Transcatheter vascular embolization, including transcatheter arterial embolization (TAE), is an invasive procedure that helps to decrease portal pressure. Percutaneous transhepatic variceal embolization (PTVE) punctures the portal vein using visualizing techniques such as B-Scan ultrasonography and causes embolization of the bleeding vessel [14,15]. It is the first-line treatment for portal hypertension and varices of the gastric fundus. It is a much simpler treatment modality in comparison to TIPS. The preferred treatment modality is the early use of TIPS in unsuccessful conservative and endoscopic therapy as a salvage measure [16-18]. 

**Figure 1:**
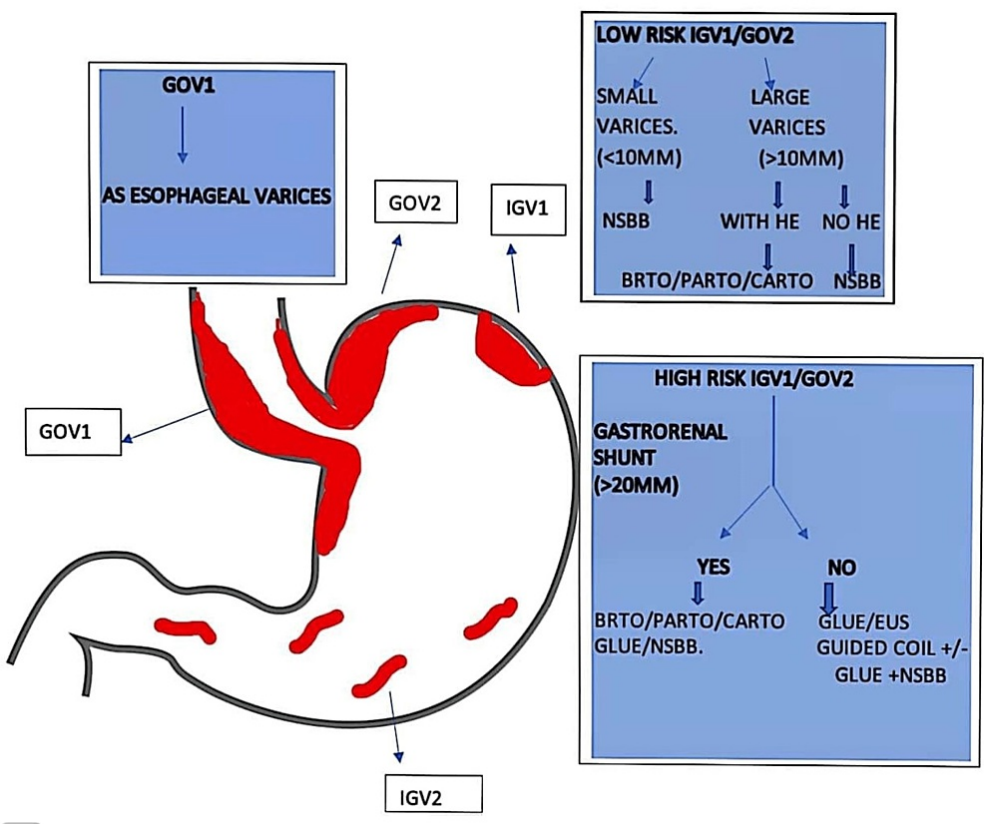
Management of gastric varices Source: Reference no. [23] GOV: Gastric variceal obturation; EVL: Endoscopic  variceal ligation; HE: Hepatic encephalopathy; NSBB: Non-selective beta blocker; CARTO: Coil-assisted retrograde transvenous obliteration; PARTO: Plug-assisted retrograde transvenous obliteration

Current guidelines suggest EVL should be performed on GVs smaller than 2 cm in diameter because ligation is done with standard rubber bands [19,20]. In contrast, larger-diameter GVs require larger detachable snares, which can increase the risk of re-bleeding. EVO is considered a more effective method of hemostasis [21,22]. All the relevant treatments discussed earlier are summarized in Figure 1. 

**Figure 2:**
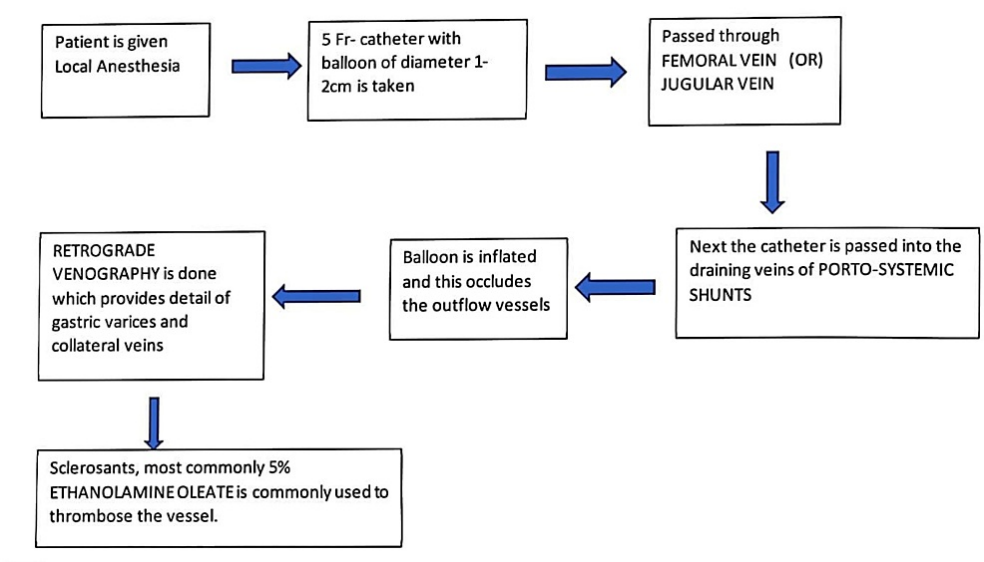
BRTO's procedure described in the flowchart BRTO: Balloon-occluded retrograde transvenous obliteration Source: Reference no. [9]

Balloon-occluded retrograde transvenous obliteration (BRTO) is a suitable method for gastric variceal bleeding (GVB) because it scleroses the GV (Figure 2) and is widely used in the East (Japan and Korea) [4,24]. In addition, balloon-occluded retrograde transvenous obliteration appears feasible and successful in patients with poor hepatic functional reserve or hemorrhagic diathesis [14]. However, whether TIPS or BRTO is more beneficial for GVB patients, especially regarding the overall survival rate, still needs to be discovered [14].

Comparison between TIPS and BRTO 

TIPS and BRTO are essential treatment modalities, with TIPS being well-known and more widely accepted. TIPS is considered more effective in decreasing the portal pressure but also stands to be more invasive and does carry a higher risk of complications [25]. TIPS significantly improves survival in cases of hepatic venous pressure gradient of more than 20 millimeters of mercury and cases of increased re-bleeding. TIPS redirect blood flow from the portal circulation to the heart via the inferior vena cava, bypassing the liver. The liver has some essential functions compromised because of this bypass leading to increased build-up of toxic metabolites such as nitrogenous products, giving rise to complications such as hepatic encephalopathy and myelopathy.

BRTO is a more straightforward and rapid procedure; it involves direct sclerosing of the bleeding vessels and does not affect the portal pressure [26]. Unlike TIPS, BRTO does not reduce the incidence of ascites as pressure build-up is not improved; thus, this may also lead to bacterial overgrowth in the peritoneum [14]. BRTO and TIPS, used together, allow the preservation of the liver’s function and synthetic capabilities. 

Gastric variceal size, volume and collaterals are essential markers for deciding which treatment modality to use and have been classified into five grades by Hirota et al. based on the gastric vasculature anatomical features and hemodynamic flow (Table 1), which was determined by gastric venography [27]. It has been reported that around 40% of cases require multiple procedures, i.e., an average of two or three procedures for successful haemostasis. These repeated procedures are deemed to be burdensome for the healthcare system and the patients [28].

**Table 1:** The table depicts classification of gastric varices Source: Reference nos. [24,27]

GRADES	GASTRIC VARICES FEATURES	COLLATERAL VEINS FEATURES
Grade 1	Well opacified	Not present
Grade 2	Contrast remained in varices for 3 min or more	Small in size, few in number
Grade 3	Contrast filled the varices partially and disappeared within 3 min	Medium to Large in size, few in number
Grade 4	Not opacified	Large in size, Many in number
Grade 5	Due to very large gastrorenal shunt with rapid blood flow, the left renal vein cannot be occluded	

Endovascular procedures such as TIPS and BRTO have become essential in controlling gastric variceal bleeding when compared to other methods, such as the endoscopic route has its limitation and seems to be less efficacious when it comes to GVB, and placement of surgical shunts is less preferred in patients themselves [29].

According to Gimm et al., the five-year survival rate post-operatively was more significant for BRTO than TIPS [30]. According to Ninoi et al., the overall survival rate after five years was notably higher for transcatheter sclerotherapy (BRTO) in Child-Pugh class A but was essentially the same in Child-Pugh classes B and C as compared to TIPS [25]. Lee et al. concluded that the three years survival rate was higher for BRTO than TIPS [31]. Choi et al. [26] and Sabri et al. [32] reported that the one-year survival rate after the procedure was longer after BRTO than TIPS. The median survival rate was 26.6 months (about two years) in patients who got BRTO and 16.6 months in patients who underwent TIPS [32,33]. BRTO has an overall better survival rate when compared to TIPS [14]. Despite the above data, early use of TIPS treatment stands as the first-line treatment for acute upper gastrointestinal bleeding, as it is reported by Garc´ıa-Pag´an et al. that the early use of TIPS helps reduce the mortality rate [34]. According to a meta-analysis study by Qi et al., TIPS with covered stents may increase survival compared to other medical/endoscopic therapies for acute bleeding [35]. As emergency procedures, both TIPS and BRTO have successfully achieved haematosis. The only difference includes the rate of complications (hepatic encephalopathy) after the procedure [14].

Advantages of BRTO

BRTO causes increased portal blood flow and causes an increase in hepatic function, and significantly improves prothrombin time [36]. BRTO may be a more effective treatment option than EIS for patients with gastric varices who have failed previous treatments and may be an excellent alternative to TIPS for patients who are not decent contenders for that procedure [37].

BRTO has been essential in significantly reducing GVs [38-42] and used prophylactically to reduce incidence [24,40,43]. It is used as an emergency treatment modality [41,44,45]. Multiple studies have shown that BRTO is more effective than endoscopic methods in achieving hemostasis and with better long-term effects [44,46,47]. It also has a slight advantage over TIPS in terms of rebleeding, hepatic encephalopathy, hepatic functional reserve, and survival [31-33]. 

Shortcomings associated with BRTO

BRTO necessitates a solid foundation in anatomy, imaging, and handling techniques. According to Wang et al., BRTO was not more challenging to complete than TIPS [48]. In addition, Othita et al. [49] reported that for all patients, splenectomy with gastric devascularization significantly increased overall survival (OS) compared to BRTO [3]. The two groups' rates of rebleeding occurrence were the same. Although in both groups liver functions improved, splenectomy with devascularization was generally more efficient than BRTO at enhancing liver health and lowering thrombocytopenia. Patients with BRTO-resistant varices that have increased input pathways, high-grade portal hypertension, and coagulation issues undergo splenectomy with devascularization [49].

Renal impairment, hemolysis, development of ascites, and oesophagal varices are some of the negative consequences of BRTO [50]. There is a significant correlation between respiratory failure, hepatic failure, portal vein thrombosis [51], and balloon rupture [52]. Additionally, it causes a postoperative rise in portal vein pressure, which increases the likelihood of esophageal varices (EV) aggravation and ascites development [53]. Additionally, it should not be used in portal and splenic vein thrombosis cases since the blood accumulation could cause splenic ischemia [12].

BRTO uses ethanolamine oleate, which can have serious side effects, including intravascular hemolysis and hemoglobinuria (49-100% of cases) [42,54] renal dysfunction [55]; allergic reactions following balloon-occluded retrograde transvenous obliteration result in cardiogenic shock (5%) [27]; also, pulmonary embolism results in pulmonary infarction [56] or acute respiratory distress syndrome due to alveolar wall edema and pulmonary congestion following endoscopic injection sclerotherapy. Some of the above problems are related to the amount of ethanolamine oleate administered [54]. 

Advancement in technologies of BRTO

Vascular plug-assisted BTRO and coil-assisted BRTO, which do not utilize an indwelling balloon catheter, are more accessible and faster than classic BRTO. When TIPS is combined with balloon-assisted antegrade BRTO, hemostasis is improved, and the risk of consequences from portal hypertension, such as refractory ascites and portal thrombosis, is reduced [57]. In contrast to traditional BRTO, selective BRTO, in which only the varices are embolized, has been shown to reduce the elevation in portal pressure and the risk of EV and ascites aggravation [53]. Therefore, this targeted approach might lessen the postoperative aggravation of EV [58]. In the study by Atsushi et al. selective BRTO observed less commonly the EV and ascites deteriorating compared to standard BRTO. Selective BRTO allows for the preservation of the significant drainage vein without obliterating it, which reduces the quantity of sclerosant needed. Compared to standard BRTO, the volume of the embolized vessel is small, and much less sclerosant is utilized overall [58]. However, a higher recurrence rate was linked to it [58]. Therefore, if selective BRTO is performed, rigorous follow-up is required to identify GV recurrence.

Foam sclerotherapy is a cutting-edge substitute for liquid sclerotherapy that lessens its side effects. The overall dose of the sclerosing agent is lower than that used for liquid sclerotherapy since the active agent is only present at the bubbles' surface, and the bubbles' center only contains gas. Due to the lesser amount of sclerosing agent that could leak into the central veins and systemic circulation, this dose reduction has increased safety [59]. Foam sclerosants tend to ascend right away into the nondependent target gastric varices ventral to the gastro-renal shunt, which can be advantageous in challenging obliteration instances where the target gastric varices cannot be reached with the catheter due to excessively tortuous veins. The foam can ascend from the more dorsal gastro-renal shunt into more ventral gastric varices and eventually trap within as an air pocket, leading to thrombosis [28]. As a result, treating gastric varices with balloon-occluded retrograde transvenous obliteration and foam sclerosant under C-arm CT guidance allows for a significant reduction in the volume of sclerosant injected and a better safety profile. Technical success (variceal thrombosis) can be accomplished in a single surgery in 95% of instances, mainly due to the physical characteristics of foam. The procedure's C-arm CT guidance is dependable for verifying that the target vessels are filled with foam till the optimum level [28]. 

Limitations of the study

BRTO is relatively a new treatment modality that has not been widely implemented yet. Due to this reason we didn't have enough literature to explore. We did not include unpublished or gray literature which might have provided more data. We excluded pediatric population and only focused on adult population with varices. There was no significant data with regards to response of treatment based on size of varices. Lack of data with regards to response & prognosis based on sub classification of Child Pugh classification. Co-morbidities were not taken into consideration.

## Conclusions

BRTO is an effective and safe treatment option for gastric varices. BRTO has been shown to have high rates of variceal obliteration and low rates of recurrence, with a low risk of major complications. Based on the available evidence, it can be concluded that balloon-occluded retrograde transvenous obliteration (BRTO) is a superior treatment modality for gastric varices when compared to other options, such as endoscopic injection sclerotherapy (EIS) and transjugular intrahepatic portosystemic shunt (TIPS). Also, BRTO is associated with higher rates of complete obliteration of varices, lower rates of rebleeding, and improved overall survival compared to EIS and TIPS. In addition, BRTO is less invasive than TIPS and has a lower risk of hepatic encephalopathy.

Furthermore, despite the potential advantages of BRTO it is important to consider patient-specific factors and individualize treatment plans accordingly. Further research is needed to elucidate the optimal treatment approach for gastric varices fully, but current evidence suggests that BRTO should be considered as a therapy in eligible patients.
